# Articulating the ultimate objectives of research capacity strengthening programmes: Why this is important and how we might achieve it.

**DOI:** 10.12688/f1000research.153447.1

**Published:** 2024-08-08

**Authors:** Justin Pulford, Meshack Nzesei Mutua, Imelda Bates, Nadia Tagoe

**Affiliations:** 1 Liverpool School of Tropical Medicine, Liverpool, UK; 2 Kwame Nkrumah University of Science and Technology, Kumasi, Ghana

**Keywords:** Research capacity strengthening; evaluation; global South

## Abstract

‘Research capacity strengthening’ (RCS) is an umbrella term that can be used to describe a wide variety of activities conducted in support of diverse objectives premised upon distinct, potentially opposing, views. Despite this, the ultimate objective of RCS activities is rarely made explicit which can be problematic when diverse objectives are possible. By ‘ultimate’ objective we are referring to the overarching (often long-term) goal an RCS initiative is intended to contribute towards (e.g. better population health) as opposed to the more immediate ‘proximate’ (often short-term) objectives of any such activity (e.g. improved capacity to undertake infectious disease research).

We argue a need for those funding, designing and implementing RCS initiatives to make clear statements as to the ultimate objective that they foresee their respective initiative contributing towards as well as the proposed pathway and associated assumptions that underlie their approach. Examples of distinct ultimate objectives for RCS initiatives are presented alongside fictitious examples of how they may be transparently reported from both a funder and implementor perspective.

Such transparency should be routine within the scope of funding calls for RCS activities (even when such activities are only a minor component of the call), subsequent applications to those calls and any description of an applied RCS activity/ies and/or the associated outcomes thereof. The process of determining one’s ultimate objective will further cause funders and actors to think through their respective initiatives more thoroughly and make informed choices and better designed RCS projects. Doing so would reduce any ambiguity associated with the use of the term ‘research capacity strengthening’ and would provide a stronger foundation for robust programme evaluation.

## Introduction

There is a long history of governments, development partners and philanthropic organisations financing research capacity strengthening (RCS) initiatives in the global South. Common examples, among many others, include scholarship schemes, infrastructure grants and collaborative research awards. The cumulative investment in RCS support within the global South has not been calculated but would certainly exceed several billion GBP and counting. As an example, the UK government and Wellcome spent £873 million between 2016-2021 on dedicated RCS initiatives in the global South with a further £1.2 billion expended on research activities with a capacity strengthening component. ^1^ Benefits of RCS investment have been widely reported in the literature ^2^^–^^4^ and gains in Southern research capacity are apparent in measures such as scientific publication output. ^5^ Nevertheless, much of this investment is based on very little evidence of the effectiveness or impact on long term outcomes. Southern research capacity remains well behind Northern standards on most metrics ^6^^,^^7^ and there is still much to be learnt about the value for money of these investments and about the pathways by which impact in the global South is achieved. ^4^

Limited understanding of RCS impact reflects, in part, a lack of sound evaluation. ^8^^,^^9^ RCS is a long-term, multi-faceted and complex undertaking and the tools to support robust, standardised evaluation are only beginning to emerge. ^10^^–^^12^ A further factor confounding evaluation efforts is a pervasive ambiguity in the use of the term ‘research capacity strengthening’ itself. RCS is not a construct in its own right; rather, RCS is an umbrella term that can be used to describe a wide variety of activities conducted in support of diverse objectives premised upon distinct, potentially opposing, views (see further below for examples). Despite this, the RCS term is often used with a false assumption of neutrality ^13^ and without a clear definition being provided. ^8^ Those definitions that are presented are highly diverse and typically process oriented, yet it is not at all apparent whether different definitions of RCS represent distinct conceptualisations of RCS as an undertaking or whether (as is more likely) they are being used with a broadly consistent (yet often ambiguously stated) meaning (see Box 1). Even the term ‘research capacity strengthening’ is often used interchangeably with the terms ‘research capacity building’ and ‘research capacity development’ ^8^ despite attempts to differentiate their meaning. ^9^^,^^14^ Without absolute transparency about the agreed explicit, ultimate objective of an RCS initiative, and the assumptions upon which it is based, then our ability to advance understanding of what works well and why in RCS practice through applied research, evaluation and shared learning is compromised. By ‘ultimate’ objective we are referring to the overarching (often long-term) goal an individual RCS initiative is intended to contribute towards (e.g. better population health) as opposed to the more immediate ‘proximate’ (often short-term) objectives of any such activity (e.g. improved capacity to undertake infectious disease research). Proximate objectives remain important; they are just not the focus of this paper.

Box 1. **Defining research capacity strengthening.**

A scoping review of health-related RCS papers published in academic journals between 2000-2016 identified a total of 172 papers of which only 19% (33/172) presented some form of RCS definition ^8^. In the 33 papers that did so, a total of 25 distinct definitions were provided. Seventeen of these 25 definitions pertained to either ‘health research capacity strengthening’ or ‘research capacity strengthening’ (or variants thereof such as ‘research capacity building’) whilst the remaining eight pertained to broader definitions of ‘capacity strengthening’ (or variants thereof) that were not specific to research. Many of these definitions emphasised RCS as a ‘process’ without specifying an objective beyond strengthened capacity, e.g.

*“Process of individual and institutional development which leads to higher levels of skills and greater ability to perform useful research.”* ^15^

*“A long-term process that requires a systematic and inter-sectoral approach to developing appropriate regulatory frameworks, building and maintaining physical infrastructure, and investing in human resources, equipment and training in an environment conducive to research commitment and institutional support.*” ^16^

In definitions where an objective was indicated, this was typically couched in terms of the capacity to identify and resolve ‘problems’, e.g.

*“The ongoing process of empowering individuals, institutions, organisations, and nations to: define and prioritise problems systematically; develop and scientifically evaluate appropriate solutions; and share and apply the knowledge generated.”* ^17^

*“Strengthening the abilities of individuals, institutions, and countries to perform research functions, defining national problems and priorities, solving national problems, utilizing the results of research in policy making and programme delivery.”* ^18^

The latter examples explicitly draw a link between research capacity and national development (as described below), although this does not then mean that other definitions are used in support of a different objective; it is simply not possible to tell unless the ultimate objective is clearly articulated.

The aim of this paper is to justify and promote the need for transparency in RCS practice in making the ultimate RCS objective(s) explicit and to provide some recommendations as to how this might be achieved. Not only will this support more effective learning and evaluation, greater clarity of purpose for any RCS activity will also help inform the design and implementation processes to ensure that they are compatible with, and contribute towards achieving, the stated objectives. To substantiate our argument, we outline three different ultimate objectives for RCS activities. We also highlight the considerable scope for distinct and potentially divergent pathways through which RCS activities might support these objectives, even when the ultimate objective is agreed, well-defined and shared. In so doing, we are not endorsing any one approach over another, we are merely demonstrating that the common term ‘research capacity strengthening’ can be used to describe a wide range of potentially divergent activities. The examples we provide should not be considered exhaustive. Further examples, with differing implications, are almost certainly possible and we would encourage others to articulate these in the public domain and to challenge and refine those presented here. We will begin with a description of what appears to be the most common ultimate objective for RCS investment in the global South: (inter) national development.

### RCS in support of international development

Much contemporary RCS practice in the global South, whether explicitly stated or not, is conducted within an international development context. By ‘international development’ we are referring to any contributory effort towards improving the health and/or development status of the intervention country. This positioning is evidenced in the two statements quoted below. The first from the United Nation’s Development Programme (UNDP) referring to the importance of capacity development more broadly (inclusive of, but not limited to research capacity) and the second from the Council on Health Research Development (COHRED) referring to a specific type of RCS (health):

*“A country’s successful development hinges on having sufficient capacity. While financial resources, including official development assistance, are vital, they are not enough to promote sustainable human development. Without supportive strategies, policies, laws and procedures, well-functioning organizations, and educated and skilled people, countries lack the foundation to plan, implement and review their national and local development strategies”.* ^19^

*“We find that essential national health research is a critical tool for equitable health and development and therefore recommend that each developing country, taking account of its own circumstances, make careful plans for and carry out sustained, long-term programs for building research capacity and conducting essential national health research.”* ^20^

The ultimate objective of any RCS initiative conducted within an international development context, then, would be a measurable improvement in a nation’s health and/or development status. However, there are diverse views on what factors, processes or systems may best drive national development which will in turn influence decisions as to what specific development objectives should be prioritised and what corresponding research capacities need to be strengthened. Thus, referencing ‘(inter) national development’ as a RCS objective remains obscure if the type of development sought, the specific development objectives and the proposed pathway towards achieving them are not articulated. For example, proponents of market economics might recognise a transition towards a knowledge-based economy as a key driver of socio-economic development and on this basis prioritise RCS in science, technology, engineering and mathematics (STEM) as essential precursors to any such transition. Others may prioritise national health research capacity on the basis that population health is fundamental to national development per se, irrespective of the underlying economic model. Alternatively, RCS initiatives may be based on achieving progress against a specific development indicator, such as one or more of the sustainable development goals (SDGs); which scientific disciplines or research fields are then prioritised for capacity strengthening would vary accordingly.

RCS interventions undertaken within an international development context may also vary in terms of design, even when prioritising the same development objective or scientific discipline. RCS initiatives have historically focused on strengthening capacity of individuals, such as researchers and research team members, as opposed to the institutions or broader research systems they work within, ^21^ yet funders are now increasingly emphasising the importance of institutional and systems level interventions. ^22^ RCS investments have also often emphasised notions of ‘research excellence’ on the premise that it is innovative, high-quality research that drives development. ^23^ However, this can result in disproportionately large investment in a smaller number of the comparatively better capacitated universities and research institutions in the global South further exacerbating local, national or regional disparities in research capacity. ^24^^–^^26^ Thus, the concept of equity has become a key consideration in the field with a view towards more power and resources being directed towards the less-well capacitated individuals, institutions, and nations across the global South. ^27^ There is limited evidence-based guidance to inform which of the many possible variations in RCS approach may be most effective in terms of supporting an international development objective whatever the specific objective might be. Rather, legitimate arguments can be made in support of multiple approaches and different RCS initiatives make different choices, even when operating within the same overarching programme. ^28^

Without clear statements as to the specific development objectives sought and the proposed pathway towards achieving these objectives, it becomes difficult to design and implement optimally conducive RCS activities and evaluate their success. To illustrate, in some of the examples presented above different scientific disciples or research fields are being prioritised for capacity strengthening (STEM vs health research vs SDG appropriate research) yet it would not be possible to make or to understand these prioritisation decisions without at least some understanding of the underlying development objective. Similarly, the approach and ethos to designing and implementing the RCS activities once the specific objective was agreed would also likely vary across these examples. Notions of attaining research excellence within the defined standards of a Northern research tradition may be highly appropriate to RCS activities that prioritise STEM subjects in support of transitioning towards a knowledge economy, yet the same approach may be less compatible with RCS activities undertaken in support of empowering local community health initiatives. No one approach is necessarily more or less appropriate than any other, nor indeed would any one approach be exclusive to any one development objective; however, clarity as to the RCS purpose in each case would likely signal the more suitable implementation approach further enhancing capacity outcomes and providing a clearer basis for subsequent evaluation.

To further complicate the situation, we now argue that RCS in the global South can also be conducted outside of an international development context altogether. We present two such possibilities: 1) RCS in support of knowledge as a common good and 2) RCS in support of decolonisation.

## RCS activities outside of an international development context

Knowledge has long been regarded as a form of common or public good ^29^ as have public Universities. ^30^ The extent to which (all) *scientific* knowledge may be considered a common good or the extent to which public universities are genuinely in the public good are open to debate. ^30^^,^^31^ However, within the parameters of these ongoing debates, it remains plausible to frame RCS investment (or at least some forms of RCS investment) as a contribution to the common good. This may overlap with an international development approach to some degree, as scientific knowledge that supports international development may also be considered a common good ^29^; yet it also encompasses scientific disciplines and research fields such as Philosophy, Theology or History that are less likely to attract funding within an international development context. The ultimate objective of any such RCS investment would not be the outcome of knowledge application (e.g. such as a development goal), but a measurable increase in the quantity, quality, diversity and/or access to (scientific) knowledge itself.

RCS investments could also potentially be framed within a decolonisation context. It is widely recognised that a consequence of the European colonial era was the disruption, degradation or destruction of traditional customs and bodies of knowledge among colonised people. ^32^ Colonisation also instituted the imposition of the coloniser’s ideals, practices and standards which ended up suppressing indigenous knowledge practices. ^33^ It would therefore be reasonable to consider strengthening capacity for research that supports the restitution of traditional knowledge bodies and/or strengthening local systems of research as conceptualised by the formerly colonised peoples or their descendants. The ultimate objective of any RCS activity in such instances would be greater recognition of indigenous knowledge systems and promotion of self-determination at local/national/regional levels regarding capacity goals, needs and the pathways towards achieving specified goals. As with the international development example, the RCS activities conducted in support of either a common good or decolonisation objective, along with the proposed pathways to impact and associated assumptions, would also be open to considerable variation.

These examples demonstrate that activities described as ‘research capacity strengthening’ can be implemented in support of diverse ultimate objectives and premised upon a multitude of legitimate, yet potentially very different, pathways to achieving their respective objective. Distinct scientific disciplines or research fields may be more or less likely to receive capacity strengthening support depending upon what the ultimate objective is and, in some cases, these prioritisation decisions could be diametrically opposed. Hence the call for greater transparency re the ultimate objectives when describing or reporting on future, current or past RCS activities. In practice, RCS programmes may have multiple or over-lapping objectives. For example, Mormina and Istratii recently argued a case for a decolonisation approach to RCS in support of international development objectives ^13^ and, as noted above, science conducted as a common good may also support development objectives in some instances. This is in no way problematic if the primary or overlapping ultimate objectives are clearly articulated. When such transparency is lacking, as argued above, it becomes difficult to evaluate the outcomes of a RCS intervention or even to design and implement an intervention in a manner optimally conducive to achieving the objective. This is analogous to any other ‘experiment’ i.e. if the ultimate objective is not clearly stated from the outset, alongside any associated assumptions as to how best to achieve that objective, then how can an optimal intervention be designed in the first instance?

## Achieving transparency in practice

To aid the appropriate interpretation of RCS activities as well as subsequent learning and evaluation, we propose that those funding, designing and implementing RCS initiatives make clear statements as to the ultimate objective that they foresee their respective initiative contributing towards. They should also describe the proposed pathway and associated assumptions that underlie their approach. Key principles that are expected to be, or were, prioritised in RCS design and implementation such as ‘excellence’, ‘equity’ or ‘collaboration’ may also be emphasised. These statements do not need to be overly complex or lengthy. To illustrate, some fictitious examples of transparent statements about the ultimate RCS objectives from both a funder and implementor perspective are presented below, along with a note on how they support transparent RCS reporting and would inform subsequent evaluation ( Box 2).

Box 2. **Fictitious examples of transparent RCS reporting.**

Example 1: RCS in support of international development

Funder statement: This programme is designed to strengthen research capacity in STEM-related disciplines in ODA-eligible countries. The programme is premised on the view that greater national research capacity in STEM-related disciplines will provide a stronger foundation for the development and uptake of innovative, locally appropriate technical solutions to priority national and regional challenges and contribute towards economic growth through innovation-led commerce and enterprise.

Implementor statement: Our project is designed to strengthen research capacity in computational modelling in [name of focal nation/s]. We will achieve this by supporting the development of a specialist ‘centre of excellence in computational modelling’ housed within the University of X and supporting them to cascade these skills to other institutions. This approach is premised on the view that the fastest and most efficient way to develop research capacity in computational modelling in [nation] is through intensive investment of time and resources within a specialist centre that can then support the development of similar expertise in other institutions throughout the country and region (i.e. a ‘hub and spoke’ model).

*Comment: The ultimate objectives (addressing priority national/regional challenges & contributing towards socio-economic development) of the RCS funding are made clear in the funder statement as is the pathway towards achieving these objectives (STEM-derived innovative, locally appropriate technical solutions with potential for commercialisation). The implementor statement makes clear the approach (investment in a computational modelling centre of excellence) with which their project will contribute towards the ultimate objective as well as the assumptions underlying this pathway (fastest way to build capacity in the short-term and efficient in the long-term when considered the’ hub’ for future ‘spokes’). Evaluation of this RCS project would therefore look for examples of technical solutions that the centre for computational modelling has developed (or is currently developing) that have addressed (or could potentially contribute towards addressing) a national/regional challenge and/or that have been (or potentially could be) commercialised.*

Example 2. RCS in support of knowledge as a common good

Funder statement: This programme is designed to strengthen research capacity in the Arts and Humanities. The programme is premised on the view that creating, and ensuring access to, new knowledge from across traditional academic disciplines is in the common good. The Arts and Humanities have been prioritised in this instance as, relative to many other academic disciplines, research funding in these fields is scarce.

Implementor statement: Our project is designed to support five early career researchers, one from each of five universities belonging to our consortium, to undertake three-year post-doctoral study within an Arts and Humanities subject of their choosing. Along with the financial resources necessary to undertake their study, each scholar will be provided expert mentorship and specialist leadership training to assist their career development and we will support the production of multiple, publicly accessible outputs (e.g. books, artwork, film) arising from their work. This approach is premised on the view that each University within our consortium should have the right to self-determine which Arts and Humanities discipline is prioritised for funding in their context. Our approach further recognises that early career researchers in the Arts and Humanities need specialist funding and guidance to develop a productive and sustainable career in what is a competitive academic environment and that the value of their work to society will be best served by ensuring that it is made readily accessible in easy to understand and engaging ways.

*Comment: The ultimate objective (contributing to the common good) of the RCS funding is made clear in the funder statement as is the premise upon which the Arts and Humanities were prioritised (neglected relative to other academic disciplines). The implementor statement makes clear the pathway (mentored and resourced early career researcher scholarships and support to produce accessible outputs) with which their project will contribute towards the ultimate objective as well as the assumptions underlying this pathway (scholars in Arts and Humanities need specialist funding and support to develop and sustain careers in a competitive environment). Evaluation of this RCS project would therefore look for examples of outputs that represent new knowledge and evidence that this knowledge has been made accessible to, and has been accessed by, the public.*

Example 3. RCS in support of decolonisation

Funder statement: This RCS programme is designed to strengthen indigenous knowledge and/or knowledge systems in [nation]. This investment is provided by [donor] in recognition that indigenous knowledge/knowledge systems are a precious national and global resource. All decisions related to how this funding will be used to support RCS within [nation], including how the notion of research is conceptualised, is for [nation] to self-determine.

Implementor statement: Our project is designed to ensure the oral histories of [a people] are recorded for posterity and made accessible for the national good. It is our belief that a full understanding of our nation’s people, our current place in the world and our future pathways is not possible without reference to our own unique histories which are at risk of being lost or neglected if not recorded and communicated in ways that everyone in our society can engage with.

*Comment: The ultimate objective (strengthen indigenous knowledge and/or knowledge systems) of the RCS funding is made clear in the funder statement as is the premise upon how the funding will be used to support this objective (for the recipient nation to self-determine). The implementor statement makes clear how the funding will be used in support of this objective (record oral histories) as well as the assumptions underlying this decision (it is in the national good to do so and a risk of oral histories being lost or neglected if the work is not undertaken). Evaluation of this RCS project would therefore look to establish that the work was self-determined independent of funder influence and that there was a measurable increase in, and/or access to or influence of, indigenous knowledge.*

These examples illustrate what should be considered a minimum acceptable level of transparency. Understanding of any RCS activity would be further enhanced by fuller descriptions of proposed impact pathways and there are widely used methods and tools for doing so, including the use of programme Theories of Change, ^34^ logic models ^35^ and even specialist frameworks intended to support better, more transparent RCS practice and evaluation. ^12^ Even basic descriptions such as those presented above would represent a considerable improvement in transparent reporting of RCS activities if they were applied in practice. Such transparency should be routine within the scope of funding calls for RCS activities (even when such activities are only a minor component of the call), subsequent applications to those calls and any description of an applied RCS activity/ies and/or the associated outcomes thereof. The process of determining one’s ultimate objective will further cause funders and actors to think through their respective initiatives more thoroughly and make informed choices and better designed RCS projects. Doing so would reduce any ambiguity associated with the use of the term ‘research capacity strengthening’ and would provide a stronger foundation for robust programme evaluation. From such a foundation, and with appropriate investment to support routine evaluation, the research community will be in a strong position to greatly accelerate our ability to understand what works well, and not so well, in our collective RCS endeavours.

### Ethics and consent

No ethics and consent required.

## Data Availability

No data are associated with this article.

## References

[1] UKCDR: *UK funding landscape for research capacity strengthening in low- and middle-income countries: briefing paper, October 2021.* London: UK Collaborative on Development Research;2021.

[2] Aiyenigba A Abomo P Wiltgen Georgi N : Enabling research capacity strengthening within a consortium context: a qualitative study. *BMJ Glob. Health.* 2022;7(6):e008763. 10.1136/bmjgh-2022-008763 35764358 PMC9240891

[3] Vicente-Crespo M Agunbiade O Eyers J : Institutionalizing research capacity strengthening in LMICs: A systematic review and meta-synthesis. *AAS Open Res.* 2020;3:43. 10.12688/aasopenres.13116.3 33215062 PMC7653640

[4] Buser JM Capellari E Wondafrash M : Unravelling the complexity of research capacity strengthening for health professionals in low- and middle-income countries: A concept analysis. *J. Adv. Nurs.* 2024. [published Online First: 20240516]. 10.1111/jan.16232 38752602

[5] Confraria H Godinho M : The impact of African science: A bibliometric analysis. *Scientometrics.* 2015;102:1241–1268. 10.1007/s11192-014-1463-8

[6] UNESCO: *UNESCO Science Report: the Race Against Time for Smarter Development.* Schneegans S STaJL, ed. Paris: UNESCO Publishing;2021.

[7] Nabyonga-Orem J Asamani J Olu O : Why are African researchers left behind in global scientific publications? A viewpoint. *Int. J. Health Policy Manag.* 2024;13:8149. 39099524 10.34172/ijhpm.2024.8149 PMC11608291

[8] Dean L Gregorius S Bates I : Advancing the science of health research capacity strengthening in low-income and middle-income countries: a scoping review of the published literature, 2000-2016. *BMJ Open.* 2017;7(12):e018718. 10.1136/bmjopen-2017-018718 29217727 PMC5728300

[9] Franzen SR Chandler C Lang T : Health research capacity development in low and middle income countries: reality or rhetoric? A systematic meta-narrative review of the qualitative literature. *BMJ Open.* 2017;7(1):e012332. 10.1136/bmjopen-2016-012332 28131997 PMC5278257

[10] Wenham C Wouters O Jones C : Measuring health science research and development in Africa: mapping the available data. *Health Res. Policy Syst.* 2021;19(1):142. 10.1186/s12961-021-00778-y 34895277 PMC8665309

[11] Khisa AM Gitau E Pulford J : A framework and indicators to improve research capacity strengthening evaluation practice. Liverpool: Liverpool School of Tropical Medicine and the African Population Health Research Centre. 2019.

[12] Mirzoev T Topp SM Afifi RA : Conceptual framework for systemic capacity strengthening for health policy and systems research. *BMJ Glob. Health.* 2022;7(8):e009764. 10.1136/bmjgh-2022-009764 35922082 PMC9353002

[13] Mormina M Istratii R : ‘Capacity for what? Capacity for whom?’ A decolonial deconstruction of research caapcity development practices in the Global South and proposal for a value-centred approach. *Wellcome Open Res.* 2021;6(129). 10.12688/wellcomeopenres.16850.1 PMC8729187 35028423

[14] Zamfir L : *Understanding capacity-building/capacity-development: A core concept of development policy.* Brussels: European Parliamentary Research Service;2017.

[15] Golenko X Pager S Holden L : A thematic analysis of the role of the organisation in building allied health research capacity: a senior managers’ perspective. *BMC Health Serv. Res.* 2012;12:276. [published Online First: 20120827]. 10.1186/1472-6963-12-276 22920443 PMC3464180

[16] Osei-Atweneboana MY Lustigman S Prichard RK : A research agenda for helminth diseases of humans: health research and capacity building in disease-endemic countries for helminthiases control. *PLoS Negl. Trop. Dis.* 2012;6(4):e1602. [published Online First: 20120424]. 10.1371/journal.pntd.0001602 22545167 PMC3335878

[17] Lansang MA Dennis R : Building capacity in health research in the developing world. *Bull. World Health Organ.* 2004;82(10):764–770. 15643798 PMC2623028

[18] Kilic B Phillimore P Islek D : Research capacity and training needs for non-communicable diseases in the public health arena in Turkey. *BMC Health Serv. Res.* 2014;14:373. [published Online First: 20140905]. 10.1186/1472-6963-14-373 25193671 PMC4165910

[19] UNDP: *Supporting Capacity Development: The UNDP Approach.* New York: United Nations Development Programme Bureau for Development Policy;2009.

[20] COHRED: *Health research: essential link to equity in development.* New York: Commission on Health Research for Development (COHRED);1990.

[21] Mormina M : Science, Technology and Innovation as Social Goods for Development: Rethinking Research Capacity Building from Sen’s Capabilities Approach. *Sci. Eng. Ethics.* 2019;25(3):671–92. [published Online First: 20180301]. 10.1007/s11948-018-0037-1 29497970 PMC6591180

[22] ESSENCE on Health Research and CCR: Effective Research Capacity Strengthening: A Quick Guide for Funders. 2023.

[23] Kraemer-Mbula E Tijssen R Wallace M : *Transforming Research Excellence: New Ideas from the Global South.* Cape Town, South Africa: African Minds;2020.

[24] Yarmoshuk A Abomo P Fitzgerald N : A mapping of health professional and post-graduate health programs in the WHO African Region. *AAS Open Res.* 2021;4(55). 10.12688/aasopenres.13320.1 PMC10891426 40078894

[25] Morel T Maher D Nyirenda T : Strengthening health research capacity in sub-Saharan Africa: mapping the 2012-2017 landscape of externally funded international postgraduate training at institutions in the region. *Glob. Health.* 2018;14(1):77. 10.1186/s12992-018-0395-0 30064479 PMC6066939

[26] Kilmarx PH Maitin T Adam T : A Mechanism for Reviewing Investments in Health Research Capacity Strengthening in Low- and Middle-Income Countries. *Ann. Glob. Health.* 2020;86(1):92. 10.5334/aogh.2941 32832386 PMC7413164

[27] Maher D Aseffa A Kay S : External funding to strengthen capacity for research in low-income and middle-income countries: exigence, excellence and equity. *BMJ Glob. Health.* 2020;5(3):e002212. [published Online First: 20200317]. 10.1136/bmjgh-2019-002212 32206346 PMC7078669

[28] Tagoe N Pulford J Kinyanjui S : A framework for managing health research capacity strengthening consortia: addressing tensions and enhancing capacity outcomes. *BMJ Glob. Health.* 2022;7(10):e009472. 10.1136/bmjgh-2022-009472 36192051 PMC9535163

[29] Stiglitz JE : Knowledge as a Global Public Good. Kaul I Grunberg I Stern M , editors. *Global Public Goods.* New York: Oxfor University Press;1999.

[30] Marginson S : Higher education and the public good. *High. Educ. Q.* 2011;65(4):411–433. 10.1111/j.1468-2273.2011.00496.x

[31] Radder H : Which Scientific Knowledge is a Common Good? *Soc. Epistemol.* 2017;31(5):431–450. 10.1080/02691728.2017.1353656

[32] Ziegler J Lehner E : Knowledge systems and the colonial legacies in African science education. *Cult. Stud. Sci. Educ.* 2018;13:1101–1108. 10.1007/s11422-017-9823-3

[33] Koum Besson ES : How to identify epistemic injustice in global health research funding practices: a decolonial guide. *BMJ Glob. Health.* 2022;7(4):e008950. 10.1136/bmjgh-2022-008950 35470130 PMC9039406

[34] Mayne J : Theory of change analysis: Building robust theories of change. *Can. J. Program Eval.* 2017;32(2):155–173. 10.3138/cjpe.31122

[35] McLaughlin J Jordan G : Using Logic Models. Newcomer K Hatry H Wholey J , editors. *Handbook of Practical Program Evaluation.* Wiley;2015; pp.62–87.

