# Supplementary material for: Articulating the ultimate objectives of research capacity strengthening programmes: Why this is important and how we might achieve it
Source: F1000Res. 2024 Aug 8;13:909. [Version 1] doi: 10.12688/f1000research.153447.1 (PMC11377929; doi:10.12688/f1000research.153447.1)
Supplement: Peer review history

# Reviewer report: Reviewer response for version 1

**DOI:** 10.5256/f1000research.168351.r313119

**Published:** 2024-09-05

**Authors:** Maru Mormina

**Affiliations:** 1 Department of Philosophy, African Centre for Epistemology and Philosophy of Science, University of Johannesbourgh, Johannesbourgh, South Africa

Thank you to the authors for such a thoughtful  commentary and their valuable attempt to bring clarity and transparency to a practice that remains poorly defined and poorly understood. Research capacity building (RCB), strengthening (RCS) or development ( RCD) are much misused and abused umbrella terms, used to encompass anything and everything  but more often associated with training and development of the research workforce (researchers but also government officials, health workers, and other actors engaged in generating and using research and evidence). This is not only confusing, it is highly reductive: developing research capacity entails far more than a transfer of technical skills, but developing often intangible competencies, including the ability and opportunities to absorb and produce the tacit knowledge that underpins much of the research and innovation process. 

 The article acknowledges this ambiguity and calls for transparency not only in what those doing or funding RCS mean by it but also what they are trying to achieve, i.e. the ultimate goal. After all, without a clear understanding of where we want to go, how do we select the route, and crucially, how do we know whether we have arrived?

 That RCS initiatives should be clear about their goals is intuitive and the authors provide good examples (quite possibly inspired by their own experience in this field) to illustrate how an explicit articulation of long term goals helps inform a more effective design of capacity building activities and contributes to a more robust monitoring and evaluation of programmes. 

 Whilst the proposition is very well-argued and difficult to disagree with, I feel there are two considerations that the authors have left unaddressed, possibly due to space constraints. Nonetheless, I think they are  important to acknowledge, reflect upon, and, if possible, discuss in future updates of the paper. 

 The first and most important consideration is complexity. Whether capacity building contributes to the ultimate goal of international development, decolonisation or the common good, these goals are  'wicked problems' for which cause-effect pathways (and their respective linear theories of change) are difficult to recognize. Wicked problems are an entangled web of issues that are part of an interconnected and interdependent system, so that fixing one part of the system will cause (often unpredictable) effects in other parts of the system. Given the wicked nature of the systems that RCS activities aim to impact, it is unclear whether we will ever be able to determine with any certainty a causal relationship between a particular capacity building activity and its ultimate goal. Evaluators, at most can evaluate the activities' contribution to proximate goals, in which case the value of articulating ultimate goals lies not so much in the evaluation of RCS but perhaps in making clear the need to approach RCS as a systemic intervention.

 This brings me to my second point. As the authors will undoubtely know, there is a great deal of wasteful fragmentation, duplication and reinventing of the wheel in international development, and capacity building is no exception. Funders are largely responsible for this, although recent years have seen more concerted efforts at integration and collaboration, with joint programmes increasingly becoming the norm. I would have liked to see this addressed in this commentary and I invite the authors to reflect on how their call to transparency with regards to the ultimate goals could help funders and RCS actors take more systemic approaches consistent with the wicked nature of the problems they are trying to solve. My personal view, perhaps biased, is that the true benefit of a more transparent articulation of the ultimate goals of RCS lies in that it exposes the wicked nature of development problems and forces actors to recognise the need for, and seek pathways to, systemic integration through collaboration.

Is the topic of the opinion article discussed accurately in the context of the current literature?

Yes

Are arguments sufficiently supported by evidence from the published literature?

Yes

Are all factual statements correct and adequately supported by citations?

Yes

Are the conclusions drawn balanced and justified on the basis of the presented arguments?

Yes

Reviewer Expertise:

research capacity development in international development, systems thinking, wicked problems, epistemology, ethics, science and technology studies.

I confirm that I have read this submission and believe that I have an appropriate level of expertise to confirm that it is of an acceptable scientific standard.

# Reviewer report: Reviewer response for version 1

**DOI:** 10.5256/f1000research.168351.r313114

**Published:** 2024-09-03

**Authors:** Elizabeth S Rose

**Affiliations:** 1 Vanderbilt University Medical Center, Nashville, USA

The authors have begun an excellent discussion about the lack of a definition for research capacity strengthening (RCS). They provided evidence of the array of RCS definitions in the academic literature as well as the number of articles about RCS that do not define the term. The authors also discuss the diverse views of 'international development,' which subtly (and not so subtly) is a root of most RCS initiatives. They present a new view on RCS in decolonization. Although RCS could be seen as another form of colonization (e.g., institutions from the 'global north' deciding who needs to be 'capacitated'), the authors propose that goals of RCS should be aimed towards "greater recognition of indigenous knowledge and promotion of self-determination at local/national/regional levels," which would promote research and knowledge independence. The authors' proposal that funders, implementers, and others define RCS by including their ultimate goal in the project will help those individuals and organizations to have more clarity about their work and provide transparency in this space. Such transparency and additional thought about RCS initiatives prior to work starting could contribute to projects that better support equity and independence.

Is the topic of the opinion article discussed accurately in the context of the current literature?

Yes

Are arguments sufficiently supported by evidence from the published literature?

Yes

Are all factual statements correct and adequately supported by citations?

Yes

Are the conclusions drawn balanced and justified on the basis of the presented arguments?

Yes

Reviewer Expertise:

education & training (including public health and medical student education; faculty development; curriculum design); decolonizing global health; research capacity strengthening

I confirm that I have read this submission and believe that I have an appropriate level of expertise to confirm that it is of an acceptable scientific standard.
